# Pseudo-Specialism

**Published:** 1882-10

**Authors:** 


### PSEUDO-SPECIALISTS.

We invite careful attention to the views of our Occasional Correspondent upon the relations subsisting between specialism and the general practice of medicine on the one hand, and the public on the other. This whole question involves issues of too grave importance to the profession to admit of thoughtless contemplation, and the suggestions contained in this note of warning should be studiously considered.

The writer has loyally pointed out a great and growing evil—a system which he appropriately denominates "pseudo-specialism," the unquestionable tendency of which is towards demoralization and degradation of an honorable calling, and the destruction of respectable, desirable and venerable usages.

We freely accord to special study and special practice too its full deserts and willingly give due credit for what it has already done, and what it will surely hereafter do in advancing and perfecting the science of medicine. Yet, it should be constantly borne in mind that the self-assumed titles which specialists arrogate to themselves, may, sometimes, imply superior qualifications, but, as captivating as they are to the public ear, they certainly do not confer extraordinary or unusual priviliges.

Our esteemed correspondent may confidently count us an active ally in the cause which he has so earnestly espoused and which he so worthily represents. For the present, however, we must refrain from a full discussion of this subject, but we shall recur to it in the near future.

